# Relationship between Hyponatremia and Peripheral Neuropathy in Patients with Diabetes

**DOI:** 10.1155/2021/9012887

**Published:** 2021-08-19

**Authors:** Yongze Zhang, Chuanchuan Li, Lingning Huang, Ximei Shen, Fengying Zhao, Cailin Wu, Sunjie Yan

**Affiliations:** ^1^Department of Endocrinology, The First Affiliated Hospital of Fujian Medical University, 20 Cha Zhong Road, Fuzhou, Fujian 350005, China; ^2^Fujian Province Clinical Research Center for Metabolic Diseases, 20 Cha Zhong Road, Fuzhou, Fujian 350005, China; ^3^Diabetes Research Institute of Fujian Province, 20 Cha Zhong Road, Fuzhou, Fujian 350005, China; ^4^Metabolic Diseases Research Institute, The First Affiliated Hospital of Fujian Medical University, 20 Cha Zhong Road, Fuzhou, Fujian 350005, China

## Abstract

**Objectives:**

Hyponatremia is a common complication of diabetes. However, the relationship between serum sodium level and diabetic peripheral neuropathy (DPN) is unknown. This study was aimed at investigating the relationship between low serum sodium level and DPN in Chinese patients with type 2 diabetes mellitus.

**Methods:**

A retrospective study was performed on 1928 patients with type 2 diabetes between 2010 and 2018. The multivariate test was used to analyze the relationship between the serum sodium level and the nerve conduction function. A restricted cubic spline was used to flexibly model and visualize the relationship between the serum sodium level and DPN, followed by logistic regression with adjustment.

**Results:**

As the serum sodium level increased, the prevalence of DPN had a reverse J-curve distribution with the serum sodium levels (69.6%, 53.7%, 49.6%, 43.9%, and 49.7%; *P* = 0.001). Significant differences existed between the serum sodium level and the motor nerve conduction velocity, sensory nerve conduction velocity, part of compound muscle action potential, and sensory nerve action potential of the participants. Compared with hyponatremia, the higher serum sodium level was a relative lower risk factor for DPN after adjusting for several potential confounders (OR = 0.430, 95%CI = 0.220–0.841; OR = 0.386, 95%CI = 0.198–0.755; OR = 0.297, 95%CI = 0.152–0.580; OR = 0.376, 95%CI = 0.190–0.743; all *P* < 0.05). Compared with low-normal serum sodium groups, the high-normal serum sodium level was also a risk factor for DPN (OR = 0.690, 95%CI = 0.526–0.905, *P* = 0.007). This relationship was particularly apparent in male participants, those aged <65 years, those with a duration of diabetes of <10 years, and those with a urinary albumin − to − creatinine ratio (UACR) < 30 mg/g.

**Conclusions:**

Low serum sodium levels were independently associated with DPN, even within the normal range of the serum sodium. We should pay more attention to avoid the low serum sodium level in patients with type 2 diabetes mellitus.

## 1. Introduction

Diabetic peripheral neuropathy (DPN), one of the most common chronic complications of diabetes, occurs in as many as 50% of patients with diabetes [1]. The most common form of DPN is distal symmetric polyneuropathy. Currently, very few drugs can alter the progression of peripheral neuropathy. Even with frequent visits to medical professionals and use of prescription medications, it turns out that the clinical treatment of DPN is often unsatisfactory. Therefore, early diagnosis and prevention are considered to be far more effective than treatment. Nerve conduction velocity (NCV) studies are used to diagnose and determine the distribution and severity of DPN, as well as identify possible subclinical lesions [2]. In the late stage, diabetic neuropathy is characterized by axonal degeneration, demyelination, and fiber loss [3]. In early diabetes, a modest decrease in NCV is seen.

Additionally, the potential risk factors for DPN need to be elucidated, although it is complex and difficult. Several common risk factors, including elevated blood glucose and glycosylated hemoglobin levels, age, extended disease duration, reduced estimated glomerular filtration rate (eGFR), obesity, hyperlipidemia [4, 5], elevated urinary albumin-to-creatinine ratio (UACR) [6], low serum albumin level, and hyperuricemia [7, 8], have been postulated. However, the more comprehensive cause of DPN remains to be elucidated. Patients with diabetes, especially elderly patients, often experience electrolyte disorders, such as hyponatremia [9]. Sodium is a vital component of the human body. The external sodium ion concentration is approximately nine times that of the inside of the neuron. The constant gradient of membrane concentration maintained by the sodium-potassium pump plays a crucial role in physiological permeation, potential transfer, and neurotransmission [10]. Early studies with small sample populations showed a highly significant relationship between serum sodium and NCV [11]. Low extracellular sodium had an adverse effect on nerve cells, such as osmotic demyelination [12], and was associated with dyskinesia in patients with Parkinson's [13]. In addition, excessive sodium intake highly correlated with macular edema in patients with type 1 diabetes [14]. However, reports on the relationship between DPN and the serum sodium level are limited. Therefore, this study was aimed at investigating the relationship between the serum sodium level and DPN to provide clues for the early screening of DPN.

## 2. Materials and Methods

### 2.1. Study Population

**Figure 1:**
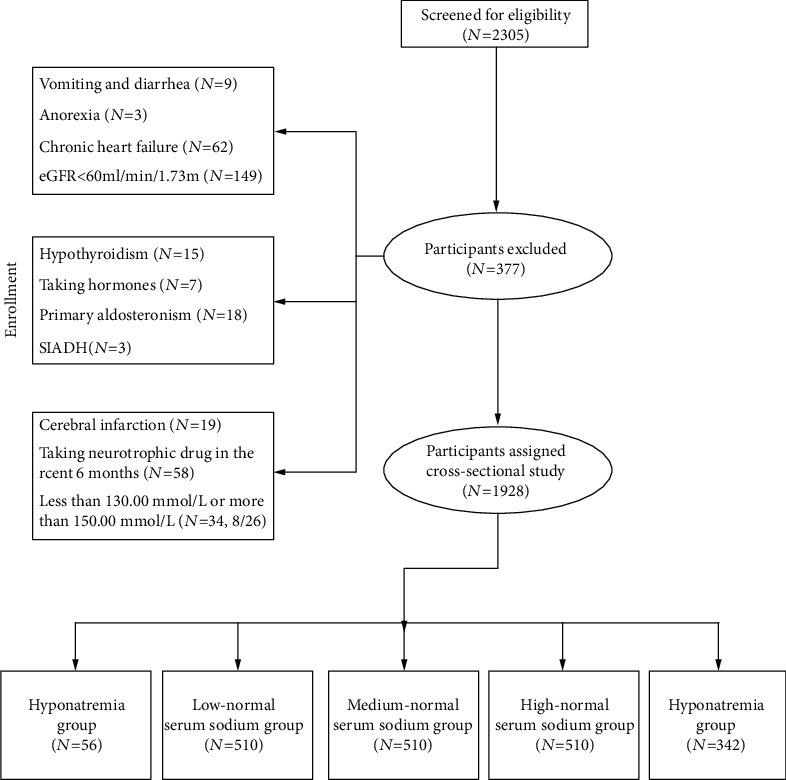
Details of excluded patients.

In this study, 1928 patients with type 2 diabetes and an average age of 60.10 years were recruited from the Endocrinology and Neurology Department at the First Affiliated Hospital of Fujian Medical University (FMU) from November 1, 2010, to January 1, 2018; the population included 1053 male and 875 female patients. No patients enrolled in the study had used neurotrophic drugs such as mecobalamin, lipoic acid, and epalrestat earlier. Patients with the following conditions were excluded:

- Type 1 diabetes, gestational diabetes, and other specific types of diabetes
- Severe and acute complications, including diabetic ketoacidosis; hyperosmolar nonketotic syndrome; gastrointestinal disturbances, such as severe vomiting and diarrhea; infection; fever with diaphoresis; eating disorders; anorexia; acute or chronic heart failure; and an eGFR of <60 mL/(min·1.73 m^2^)
- Other neurological lesions, such as chronic inflammatory response neuropathy, single neuropathy, demyelinating neuropathy, and neuropathy caused by hypothyroidism
- Taking drugs that can cause neurotoxicity, such as hormones and chemotherapy drugs
- Accompanying diseases that affect serum and/or urine sodium levels, such as primary aldosteronism, Cushing syndrome, Addison's disease, syndrome of inappropriate antidiuretic hormone secretion, and cerebral infarction
- Patients with a recent history of sodium supplementation
- Patients with corrected blood sodium less than 130.00 mmol/L or more than 150.00 mmol/L (Figure 1). The study was approved by the ethics committee of the First Affiliated Hospital of FMU, MRCTA, and ECFAH of FMU [2017]131, and written informed consent was obtained from the patients

### 2.2. Diagnostic Criteria

The participants were diagnosed with diabetes mellitus according to the criteria provided by the World Health Organization in 1999 [8]. The corrected blood sodium level of each patient was also calculated according to the blood glucose level: corrected serum sodium = serum sodium + 0.024(blood glucose × 18–100) [15]. The corrected serum sodium level was used instead of the directly measured blood sodium level. The normal blood sodium level was defined as 135.00–145.00 mmol/L, while hyponatremia and hypernatremia were defined as the level of <135.00 mmol/L and >145.00 mmol/L, respectively [16, 17]. Hypertension was defined as a systolic blood pressure ≥ 140 mmHg or diastolic blood pressure ≥ 90 mmHg. Patients actively taking antihypertensive drugs were also classified as hypertensive [18]. Atherosclerosis was defined as the protrusion of plaques into the lumen of the echo structure, protrusion of plaques into the lumen of a vessel with abnormal blood flow, or an intimal − medial thickness ≥ 1.3 mm [19]. The ischemic cardiovascular disease (ICVD)% was estimated based on the 10-year ICVD risk assessment method for Chinese people [20].

### 2.3. Clinical Measurements

Information regarding patient demographic characteristics, disease duration, lifestyle, medical history, and drug use history was obtained from medical records. All patients underwent a physical examination that included height, weight, blood pressure, and a neurological examination. Blood pressure was measured after 15 min rest. Body weight and height were measured with the patient barefoot and wearing light clothes. The body mass index of each patient was also calculated as BMI = weight (kg)/height^2^ (m^2^).

### 2.4. Biochemical Indices

Blood was obtained from the antecubital vein in all participants between 8.30 and 10.00 a.m. Laboratory tests were done to evaluate the concentration of electrolytes, including sodium, potassium (selective electrode method, Roche), and other biochemical parameters of each patient. All patients completed the electrolyte detection in 10 h of fasting to reduce the impacts of medications and foods. The interassay coefficient of variation for serum sodium was 1%. The estimated glomerular filtration rate (eGFR) = 186 serum creatinine^−1.154^ × age^−0.203^ (×0.742 female) [21] of each patient was also calculated. Furthermore, the UACR of each patient was determined in mg/g [22].

### 2.5. Evaluation of Neuropathy

#### 2.5.1. Neurological Examination

Symptoms of somatic neuropathy were documented, including numbness, burning, deep aching, and unsteadiness in walking. Neurological examinations were completed by the same experienced doctor using age-related evaluation criteria according to standard operations. During the neurological examination, touch sensation was tested using a 10 g monofilament, pain sensation was tested using a pin, reflexes were tested using a tendon hammer, and vibration sensation was tested using a standard 128 Hz tuning fork. Neurological score, neurological reflex score, and sensory function score were recorded using a 2002 Toronto Clinical Scoring System (TCSS) [23].

#### 2.5.2. Nerve Conduction Study

The nerve conduction study of each participant was determined using EMG (Danish Weidi Company, Keypoint). The body temperature of the participants ranged from 30 to 32°C. The patients were subjected to unilateral limb nerve conduction function tests. Then, the median-nerve and ulnar-nerve motor nerve conduction velocity (MCV), sensory nerve conduction velocity (SCV), tibial-nerve and peroneal-nerve MCV, and superficial peroneal-nerve and sural-nerve SCV of each patient were recorded. The corresponding amplitudes of these variables (compound muscle action potential (CMAP)/sensory nerve action potential (SNAP)) were also determined. According to the reference provided by Tang et al. [2, 24] in 1984, nerve conduction slowing was defined as nerve conduction 20% slower than that of the normal average reference value or the occurrence of two or more nerve conduction abnormalities.

#### 2.5.3. DPN Diagnosis

The diagnosis of DPN was based on the criteria proposed by an International European and North American Expert Committee. DPN was defined as patients with diabetes (having or not having clinical symptoms and signs) who had abnormal NCV, including both diagnosis and subclinical DPN [25].

#### 2.5.4. Statistical Analysis

Continuous variables were expressed as mean ± standard deviation (SD) or median (interquartile range). A chi-square test for comparing distribution was performed. Analysis of variance and Kruskal-Wallis tests were performed to determine the differences in Gaussian variables and non-Gaussian variables. *Post hoc* least significant difference test and Nemenyi test were alternative methods for further pairwise multiple comparisons to locate the source of significance. A multivariate test was used to analyze the relationship between the serum sodium level and the nerve conduction function. Furthermore, logistic regression and forest maps were used to analyze the relationship between DPN and the serum sodium level. Receiver operating characteristic (ROC) curves were configured to establish the cutoff points of the serum sodium level that optimally predicted DPN. Restricted cubic spline was used to flexibly model and visualize the relationship between the serum sodium level and DPN, and an average serum sodium level of 140 mmol/L [26] served as a reference without adjustment. Statistical significance was determined with *P* < 0.05. Statistical analyses were performed using the R software, version 4.0.4.

## 3. Results

### 3.1. Study Population Characteristics

Of the 1928 participants with type 2 diabetes mellitus, 56 presented with hyponatremia, 1530 presented with normal serum sodium levels, and 342 presented with hypernatremia. In addition, 960 of the 1928 participants were diagnosed with DPN. The patients were divided into three groups based on the diagnostic criteria, including hyponatremia, hypernatremia, and normal serum sodium groups. The normal serum sodium group was further divided into three groups (tertile 1: 135.0–141.5 (*n* = 510); tertile 2: 141.5–143.0 (*n* = 510); tertile 3: 143.0–145.00 (*n* = 510)) (Table 1). Significant changes were observed in sex, BMI, smoking, drinking history, diuretic use, oral hypoglycemic drug use, FPG, eGFR, Cr level, UACR, and TCSS. Within the normal serum sodium group, the low-normal subgroup exhibited a relatively high DPN detection rate (low-normal subgroup: 53.7%; medium-normal subgroup: 49.6%; and high-normal subgroup: 43.9%; *P* < 0.01). In addition, the hyponatremia group exhibited a higher DPN detection rate compared with the low-normal group (*P* < 0.05). Details of pairwise multiple comparisons are shown in Table 1.

### 3.2. Changes in Nerve Conduction Function by Varying the Serum Sodium Level

As shown in Table 2, the motor and sensory nerve CV and ulnar and sural-nerve SNAP increased with the increase in the serum sodium level, with some statistically significant differences among different groups.

Tibial and peroneal-nerve CMAP and superficial peroneal-nerve SNAP increased and then decreased. In the normal serum sodium group, the low-normal group exhibited a lower MCV, ulnar-nerve SCV, and superficial peroneal-nerve SCV compared with the high-normal group (*P* < 0.05). However, no significant differences were observed in CMAP and SNAP of the normal serum sodium groups. The NCV, peroneal-nerve CMAP, tibial-nerve CMAP, ulnar-nerve SNAP, superficial peroneal-nerve SNAP, and sural-nerve SNAP were lower in the hyponatremia group than in the low-normal group (*P* < 0.05). Meanwhile, a trend analysis was performed, as shown in Table 2.

### 3.3. Relationship between Serum Sodium Level and DPN

**Figure 2:**
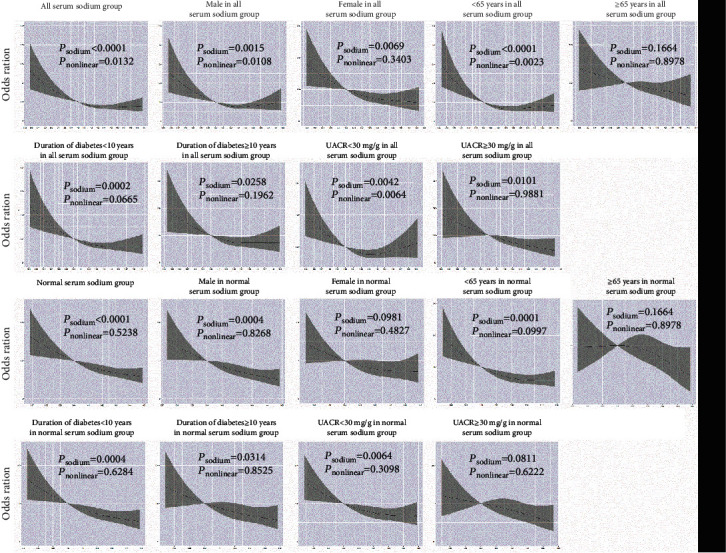
Relationship between serum sodium level and diabetic peripheral neuropathy. Restricted cubic splines were used to flexibly model and visualize the relationship between the serum sodium level and DPN. The risk of DPN was relatively flat until around 140 mmol/L of the serum sodium level and then started to increase rapidly forward and afterward (*P* for nonlinearity <0.05) in all serum sodium groups, especially in male patients, those aged <65 years, and those with UACR < 30 mg/g. However, a nonlinear trend was not observed in normal serum sodium group and its subgroups. The average serum sodium level of 140 mmol/L serves as a reference.

As shown in Figure 2, we used restricted cubic splines to flexibly model and visualize the relationship between the serum sodium level and DPN. The risk of DPN was relatively flat until around 140 mmol/L of the serum sodium level and then started to increase rapidly forward and afterward (*P* for nonlinearity <0.05) in all serum sodium groups, especially in male participants, those aged <65 years, and those with UACR < 30 mg/g. However, a nonlinear trend was not observed in the normal serum sodium group and its subgroups. It suggested that both hyponatremia and hypernatremia might increase the risk of DPN. A reverse J-curve distribution was observed between the risk of DPN and the serum sodium concentration.

**Figure 3:**
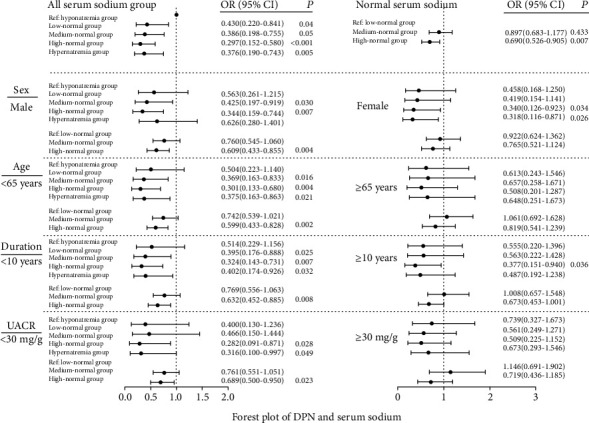
Plot of diabetic peripheral neuropathy and serum sodium level. (a) All serum sodium group and normal serum sodium group. Adjusted for age, sex, duration of diabetes, BMI, systolic blood pressure, diastolic blood pressure, HbA1c, eGFR, serum kalemia, hypotensive drugs (*β*-blocker, CCB, ACEI, and ARB), statins, hypoglycemic drugs, insulin use, smoking, drinking, and hypertension. (b) Subgroup analysis of all serum sodium group and normal serum sodium group. Adjusted for hypotensive drugs (*β*-blocker, CCB, ACEI, and ARB), statins, hypoglycemic drugs, insulin using, smoking, and drinking.

In the whole-group analysis, patients were divided into five groups according to the serum sodium level. Multiple logistic regression analyses showed that, compared with other higher serum sodium levels, hyponatremia was associated with DPN after adjusting for age, sex, duration of diabetes, BMI, systolic blood pressure, diastolic blood pressure, HbA1c, eGFR, serum kalemia, hypotensive drugs (*β*-blocker, CCB, ACEI, and ARB), statins, hypoglycemic drugs, insulin use, smoking, drinking, and hypertension (OR = 0.430, 95%CI = 0.220–0.841, *P* = 0.014; OR = 0.386, 95%CI = 0.198–0.755, *P* = 0.005; OR = 0.297, 95%CI = 0.152–0.580, *P* < 0.001; OR = 0.376, 95%CI = 0.190–0.743, *P* = 0.005, respectively) (Figure 3(a)). In all serum sodium groups, no significant relationship was detected between the subgroups of patients with diabetes aged ≥65 years or those with UACR ≥ 30 mg/g.

In the normal serum sodium group analysis, a fully adjusted logistic regression demonstrated that compared with the low-normal serum sodium level, the high-normal serum sodium level was a relatively lower risk factor of DPN (OR = 0.690, 95%CI = 0.526–0.905, *P* = 0.007) (Figure 3(a)). This relationship was particularly apparent in male participants (OR = 0.609, *P* = 0.004), those aged <65 years (OR = 0.599, *P* = 0.002), those with the duration of diabetes < 10 years (OR = 0.632, *P* = 0.008), and those with UACR < 30 mg/g (OR = 0.689, *P* = 0.023) (Figure 3(b)). The optimal serum sodium cutoff points (142.6 mmol/L) were obtained from the ROC curves.

## 4. Discussion

The present study demonstrated that patients with hyponatremia and those with low-normal serum sodium levels exhibited relatively high rates of DPN detection and relatively low NCV and amplitude. In addition, the serum sodium level was independently associated with the DPN detection rate after adjusting for several potential confounders. This relationship was particularly apparent in patients with the duration of diabetes < 10 years and UACR < 30 mg/g. This trend was also apparent within the normal serum sodium groups.

We demonstrated that patients with lower serum sodium levels were more likely to have DPN. Hospitalized patients often experience electrolyte disorders [27]. Hyponatremia and hypernatremia are the most common electrolyte disorders [28]. Sodium is a vital component of the human body. Serum sodium is closely related to hypertension [29], renal function [30, 31], fractures [32], and insomnia [33]. Hyponatremia is an independent risk factor for diabetes mellitus [28]. Otherwise, low extracellular sodium causes adverse effects in neuronal cells. Osmotic edema can also increase neuronal excitability through the activation of N-methyl-d-aspartate receptors [34], which may accelerate the development of dyskinesia [35]. Another study clarified that the serum sodium level was inversely associated with dyskinesia in patients with Parkinson's disease [13]. Currently, no studies correlating the serum sodium level with DPN in patients with type 2 diabetes mellitus are available. However, considering the important role of sodium in the central nervous system and diabetes mellitus, we do believe that the relationship exists between abnormal serum sodium levels and DPN. An early study with small sample populations demonstrated that serum sodium, but not acute blood glucose, levels had a highly significant relationship with NCV [11]. In our study, we demonstrated that hyponatremia was associated with a higher incidence of DPN and decreased NCV and amplitude. This trend, except for amplitude, was also apparent within the normal serum sodium groups, implying that hyponatremia might be a biomarker, rather than a cofounder. Furthermore, this relationship was particularly apparent in male patients with diabetes, those with duration of diabetes < 10 years, and those with UACR < 30 mg/g. In our opinion, this might be due to fewer effects of other complicated factors in these groups, which made this relationship much more obvious.

Several mechanisms might explain the relationship between DPN and hyponatremia. Sodium is a vital osmotic active solute in the extracellular compartment [36]. The sodium concentration was regulated by many factors, and it was important to maintain permeation and electrochemical gradient across cell membranes. The constant concentration gradient across the membrane played a crucial role in cell volume control, glucose transport and membrane potential, pH regulation, and neurotransmission [37]. Low extracellular sodium had an adverse effect on nerve cells, leading to osmotic demyelinating syndrome (ODS) [12]. Another explanation might be that hyponatremia could lead to a loss of excitatory neurotransmitters and transmission delays in the action potential of motor neurons [38]. The movement of sodium ions into the axon was responsible for generating the action potential [11]. The pathogenesis of DPN significantly correlated with cell electrophysiology changes, Na-K ATP enzyme dysfunction, nerve cell membrane hypoxia, cell swelling and rupture, and neuronal apoptosis [39, 40]. It was not surprising that the changes in extracellular sodium could modify nerve conduction. Otherwise, low serum sodium could lead to the functional decline of islet cells, increase blood glucose [41], and eventually cause neuropathy. We proposed the hypothesis that a relationship existed between DPN and serum sodium levels. Nevertheless, the finding of a relationship between serum sodium and DPN still needs confirmation from further studies.

Both hyponatremia and hypernatremia were associated with increased mortality [42]. Slight increases or decreases in serum sodium levels may be related to impaired neuromotor function [43]. However, the relationship between the serum sodium level and the DPN detection rate in the high-normal serum sodium group did not vary significantly from that in the hypernatremia group. Hence, a reverse J-curve distribution was observed between the risk of DPN and the serum sodium level. Previous studies showed that patients with hypernatremia presented with elevated plasma osmotic pressure, cell dehydration, and vascular endothelial injury, which could induce an inflammatory response and further exacerbate the progression of diabetes [44]. Excessive sodium also led to axonal degeneration in inflammatory demyelinating disease [45]. Patients with severe comorbidity and those with the highest recorded serum sodium and severe hyponatremia (>150 mmol/L or <130 mmol/L) [46] were excluded from this study, limiting the degree of nerve damage exhibited by the participants. We also excluded some complex diseases that might cause hypernatremia and hyponatremia. The abnormal serum sodium level might be attributed to diabetes mellitus and diuretics [28].

This study had several limitations. First, relatively few patients presented with hyponatremia. Also, patients' sodium intake and VitB levels were not recorded, which might affect the development of DPN. Second, our study design did not allow us to evaluate the causes of hyponatremia. In addition, as previously stated, the study was not designed to evaluate factors leading to this relationship. Although a role for hyponatremia in DPN is biologically plausible, we could not determine from our data whether the relationship between hyponatremia and DPN reflected a direct effect of hyponatremia, a surrogate marker for underlying comorbidities or reason for DPN, or both. Moreover, only one single measurement of fasting electrolytes was taken, which might not be an accurate estimation of the serum sodium level. The present study reported that a relative protective effect of higher-normal serum sodium concentrations was observed in patients with diabetes. However, the discrepancy in the DPN detection rate was not observed in the high-normal serum sodium group and the hypernatremia group. No survival advantage was noted once the serum sodium reached 145 mmol/L [47], and hypernatremia might cause other chronic diseases and more serious public health problems [48].

## 5. Conclusions

We must acknowledge that hyponatremia and low-normal serum sodium levels may serve as surrogate markers of DPN, treatment, or case mix. However, we believe that the mild abnormal serum sodium level should not be neglected. Since even minor serum sodium disturbances are associated with mortality, patient outcomes can be significantly improved by frequently monitoring electrolytes and discontinuing drugs with adverse effects when necessary [28]. Patients with lower sodium levels require particular care. Further studies are needed to understand the factors leading to this prognostic relationship and the potential benefit from therapeutic strategies aimed at this metabolic disturbance.

## Figures and Tables

**Table 1:** Demographic and clinical characteristics of study participants.

	Corrected serum sodium (mmol/L)					*P*
	Hyponatremia 130.0–135.0 (*n* = 56)	Low-normal sodium 135.0–141.5 (*n* = 510)	Medium-normal sodium 141.5–143.0 (*n* = 510)	High-normal sodium 143.0–145.00 (*n* = 510)	Hypernatremia 145.0–150.0 (*n* = 342)	
Corrected serum sodium (mmol/L)	133.7 ± 1.5	140 ± 1.2	142.3 ± 0.4	143.9 ± 0.5	146.3 ± 1.3	<0.001
Serum sodium (mmol/L)	132.6 ± 1.5	138.6 ± 1.9	140.9 ± 1.6	142.5 ± 1.6	144.6 ± 2.0	<0.001
Age (year)	61.7 ± 12.7	59.6 ± 12.7	59.3 ± 11.7	60.5 ± 11.6	60.9 ± 11.2	0.186
Male, *n* (%)	35 (62.5)	303 (59.4)	292 (57.3)	273 (53.5)	150 (43.9)^d^	<0.001
Duration of diabetes (year)	9 (3.3–11.8)	7 (2–10)	7 (3–10)	8 (3–12)	8 (3–12)	0.105
BMI (kg/m^2^)	22.7 ± 4.8	24.6 ± 3.7^a^	24.7 ± 3.6	24.5 ± 3.5	24.3 ± 3.7	0.004
Smoking, *n* (%)	18 (32.1)	141 (27.6)	139 (27.3)	124 (24.3)	63 (18.4)^d^	0.013
Drinking, *n* (%)	6 (10.7)	59 (11.6)	70 (13.7)	49 (9.6)^c^	23 (6.7)	0.021
Hypertension, *n* (%)	28 (50)	255 (50)	244 (47.8)	259 (50.8)	185 (54.1)	0.514
RASS-blocker, *n* (%)	16 (28.6)	135 (26.5)	105 (20.6)	115 (22.5)	71 (20.8)	0.128
*β*-Blocker, *n* (%)	3 (5.4)	34 (6.7)	34 (6.7)	41 (8)	21 (6.1)	0.803
CCB, *n* (%)	13 (23.2)	128 (25.1)	114 (22.4)	137 (26.9)	97 (28.4)	0.305
Diuretic, *n* (%)	8 (14.3)	35 (6.9)^a^	25 (4.9)	23 (4.5)	17 (5)	0.022
Statins, *n* (%)	7 (12.5)	45 (8.8)	40 (7.8)	58 (11.4)	38 (11.1)	0.255
OAD, *n* (%)	47 (83.9)	384 (75.3)	419 (82.2)^b^	420 (82.4) ^b^	280 (81.9)	0.021
Insulin use, *n* (%)	25 (44.6)	187 (36.7)	202 (39.6)	191 (37.5)	136 (39.8)	0.672
SBP (mmHg)	138.5 ± 22.2	136.8 ± 20.5	136.9 ± 19.1	136.7 ± 20.6	139.9 ± 20.6	0.140
DBP (mmHg)	75.9 ± 9.4	78.9 ± 11.3	78.5 ± 11.1	78.8 ± 11.9	77.9 ± 10.4	0.275
HbAlc (%)	9 ± 2.9	9.3 ± 2.6	9.1 ± 2.3	9.2 ± 2.4	9.1 ± 2.4	0.443
FPG (mmol/L)	8.1 ± 3.6	8.8 ± 3.5	8.8 ± 3.6	8.7 ± 3.5	9.4 ± 4.2^d^	0.045
TCH (mmol/L)	4.33 ± 1.56	4.75 ± 1.54	4.63 ± 1.26	4.67 ± 1.11	4.67 ± 1.3	0.206
TG (mmol/L)	1.2 (0.8–1.6)	1.4 (0.9–2.1)	1.4 (1–2.1)	1.4 (1–2.1)	1.4 (1–2.1)	0.050
HDL-C (mmol/L)	1.17 ± 0.44	1.11 ± 0.36	1.1 ± 0.33	1.12 ± 0.31	1.15 ± 0.35	0.270
LDL-C (mmol/L)	2.64 ± 1.42	2.88 ± 1.14	2.89 ± 1.06	2.87 ± 0.93	2.91 ± 1.03	0.538
Serum kalium (mmol/L)	4.01 ± 0.48	4.04 ± 0.46	4.02 ± 0.44	4.01 ± 0.46	3.98 ± 0.52	0.420
eGFR (mL/(min·1.73m^2^))	96.6 ± 33.3	105 ± 32^a^	109.1 ± 35.7	105.4 ± 28.7	103.2 ± 28.4	0.034
Cr (mmol/L)	71.6 ± 28.8	64.4 ± 27^a^	61.7 ± 20.2	62 ± 19.6	64.1 ± 26.8	0.017
UACR (mg/g)	34.8 (8.5–187)	12.8 (6.2–63.2)^a^	11.7 (6.2–72.6)	12.7 (6.2–38.5)	14.5 (7.3–58)^d^	0.017
Atherosclerosis, *n* (%)	23 (41.1)	137 (26.9)	149 (29.2)	151 (29.6)	102 (29.8)	0.260
Left ABI	1.06 ± 0.16	1.05 ± 0.13	1.07 ± 0.13	1.06 ± 0.11	1.09 ± 0.42	0.302
Right ABI	1.05 ± 0.14	1.09 ± 0.43	1.11 ± 0.6	1.07 ± 0.1	1.09 ± 0.27	0.577
ICVD %	10 (3.3–19)	10 (3–16)	8 (2–16)	8 (3–16)	8 (3–14)	0.222
Toronto Clinical Scoring System Score (TCSS)	2.5 (0–7)	1.0 (0–5.5)^a^	2.0 (0–6)	1.0 (0–5.0)^c^	2 (0–6)^d^	0.025
DPN, *n* (%)	39 (69.6)	274 (53.7)^a^	253 (49.6)^a^	224 (43.9)^ab^	170 (49.7)^a^	0.001

ABI: ankle brachial index; BMI: body mass index; CCB: calcium channel blockers; Cr: serum creatinine; eGFR: estimated glomerular filtration rate; FPG: fasting plasma glucose; HbAlc: glycosylated hemoglobin; HDL-C: high-density lipoprotein cholesterol; ICVD: 10-year risk of ischemic cardiovascular disease; LDL-C: low-density lipoprotein cholesterol; OAD: oral antidiabetic agents; TCH: cholesterol; TG: triglyceride; UACR: urinary albumin-to-creatinine ratio. *Post hoc* analysis: ^a^compared with the hyponatremia group, *P* < 0.05; ^b^compared with the low-normal sodium group, *P* < 0.05; ^c^compared with the medium-normal sodium group, *P* < 0.05; ^d^compared with the high-normal sodium group, *P* < 0.05.

**Table 2:** Nerve conduction velocity and nerve conduction amplitude of the different serum sodium groups.

	Serum sodium (mmol/L)					*P* value	*P* value for trend analysis	
	Hyponatremia 130.0–135.0 (*n* = 56)	Low-normal sodium 135.0–141.5 (*n* = 510)	Medium-normal sodium 141.5–143.0 (*n* = 510)	High-normal sodium 143.0–145.00 (*n* = 510)	Hypernatremia 145.0–150.0 (*n* = 342)		Linear term	Quadratic term
Median-nerve MCV (m/s)	49.73 ± 5.79	52.12 ± 5.33^a^	52.33 ± 6.35^a^	52.97 ± 5.97^ab^	52.80 ± 5.71	0.001	<0.001	NC
Ulnar-nerve MCV (m/s)	49.49 ± 7.40	52.45 ± 7.12^a^	53.70 ± 6.49^ab^	53.66 ± 6.13^ab^	53.70 ± 6.21^ab^	<0.001	<0.001	NC
Tibial-nerve MCV (m/s)	39.13 ± 7.90	42.86 ± 5.38^a^	43.67 ± 5.23^ab^	43.91 ± 5.15^ab^	43.64 ± 5.08^b^	<0.001	<0.001	NC
Peroneal-nerve MCV (m/s)	39.15 ± 7.81	41.84 ± 6.75^a^	42.88 ± 5.51^b^	43.06 ± 5.08^ab^	43.13 ± 5.34^ab^	<0.001	<0.001	NC
Median-nerve SCV (m/s)	45.21 ± 10.06	49.35 ± 8.80^a^	49.50 ± 8.62^a^	49.71 ± 9.05^a^	50.08 ± 8.01^a^	0.002	<0.001	NC
Ulnar-nerve SCV (m/s)	46.87 ± 8.63	51.13 ± 8.39^a^	52.07 ± 7.02^a^	52.00 ± 8.34^ab^	52.63 ± 6.73^ab^	<0.001	<0.001	NC
Superficial-nerve SCV (m/s)	45.22 ± 10.65	48.62 ± 13.06^a^	49.24 ± 11.92^a^	49.57 ± 13.09^ab^	49.31 ± 13.11^a^	<0.001	0.027	NC
Sural-nerve SCV (m/s)	41.30 ± 13.36	48.38 ± 10.57^a^	49.05 ± 10.46^a^	48.56 ± 12.00^a^	49.18 ± 10.22^a^	<0.001	<0.001	NC
Median-nerve CMAP (mv)	11.99 ± 4.50	12.68 ± 4.19	12.73 ± 4.12	12.49 ± 3.62	12.28 ± 4.03	0.339	NC	NC
Ulnar-nerve CMAP (mv)	11.45 ± 3.18	11.77 ± 3.21	11.73 ± 3.04	11.97 ± 2.96	12.02 ± 3.10	0.450	NC	NC
Tibial-nerve CMAP (mv)	8.30 ± 5.26	10.40 ± 5.32^a^	10.56 ± 5.66^a^	10.66 ± 5.05^a^	10.22 ± 5.02^a^	0.020	NC	0.002
Peroneal-nerve CMAP (mv)	4.46 ± 3.24	6.34 ± 3.77^a^	6.41 ± 3.65^a^	6.71 ± 3.86^a^	6.19 ± 3.57^a^	<0.001	NC	<0.001
Median-nerve SNAP (*μ*v)	14.42 ± 8.99	17.85 ± 10.75	17.92 ± 10.69	17.99 ± 9.94	17.72 ± 10.22	0.121	NC	NC
Ulnar-nerve SNAP (*μ*v)	8.93 ± 6.43	10.36 ± 5.55^a^	10.73 ± 6.24^a^	10.99 ± 5.65^a^	11.81 ± 7.98^ab^	0.001	0.001	NC
Superficial-nerve SNAP (*μ*v)	13.04 ± 14.07	16.09 ± 12.13^a^	16.68 ± 12.34^a^	16.13 ± 11.32^a^	15.74 ± 10.90^a^	0.034	NC	0.040
Sural-nerve SNAP (*μ*v)	9.13 ± 7.61	11.40 ± 7.71^a^	11.82 ± 7.81^a^	12.20 ± 7.83^a^	12.21 ± 8.37^a^	0.013	0.005	NC

CMAP: compound muscle action potential; MCV: motor nerve conduction velocity; SCV: sensory nerve conduction velocity; SNAP: sensory nerve action potential. *Post hoc* analysis: ^a^compared with the hyponatremia group, *P* < 0.05; ^b^compared with the low-normal sodium group, *P* < 0.05; ^c^compared with the medium-normal sodium group, *P* < 0.05; ^d^compared with the high-normal sodium group, *P* < 0.05. NC: nonconformance.

## Data Availability

The dataset used to support the findings of this study is available from the corresponding author upon request.
